# Effects of Air Cavity in Dynamic Pressure Sensors: Experimental Validation

**DOI:** 10.3390/s20061759

**Published:** 2020-03-22

**Authors:** Qian Dong, Xiaolei Song, Haijun Liu

**Affiliations:** Department of Mechanical Engineering, Temple University, Philadelphia, PA 19122, USA; qian.dong@temple.edu (Q.D.); xsong@temple.edu (X.S.)

**Keywords:** acoustic–structural interaction, pressure sensors, modal analysis, experimental design

## Abstract

An air-backed diaphragm is the key structure of most dynamic pressure sensors and plays a critical role in determining the sensor performance. Our previous analytical model investigated the influence of air cavity length on the sensitivity and bandwidth. The model found that as the cavity length decreases, the static sensitivity monotonically decreases, and the fundamental natural frequency shows a three-stage trend: increasing in the long-cavity-length range, reaching a plateau value in the medium-cavity-length range, and decreasing in the short-cavity-length range, which cannot be captured by the widely used lumped model. In this study, we conducted the first experimental measurements to validate these findings. Pressure sensors with a circular polyimide diaphragm and a backing air cavity with an adjustable length were designed, fabricated, and characterized, from which the static sensitivities and fundamental natural frequencies were obtained as a function of the cavity length. A further parametric study was conducted by changing the in-plane tension in the diaphragm. A finite element model was developed in COMSOL to investigate the effects of thermoviscous damping and provide validation for the experimental study. Along with the analytical model, this study provides a new understanding and important design guidelines for dynamic pressure sensors with air-backed diaphragms.

## 1. Introduction

For dynamic pressure sensors, the transduction method could be piezoelectric [1,2], piezoresistive [3], optical [4,5], or capacitive [6,7,8], but the key component of most sensors is a flexible diaphragm backed by an air cavity. In light of the recent trend of miniaturizing sensors such that they are more portable, integrated, and inexpensive [9,10], a fundamental question needs to be investigated in terms of how the miniaturization affects the sensor performance, such as the sensitivity and bandwidth.

To account for the effects of the air cavity, the most commonly used model is a lumped element model, where the diaphragm is described by a mass–spring–damper system and the backing air cavity acts as an effective spring [11]. According to this single-degree-of-freedom (SDOF) model, as the length of the backing cavity decreases, the “air spring” becomes stiffer and therefore the effective stiffness of the diaphragm increases, leading to a reduced mechanical sensitivity and increased natural frequency. However, this model breaks down when the cavity length is smaller than some critical value, as explained below.

**Figure 1:**
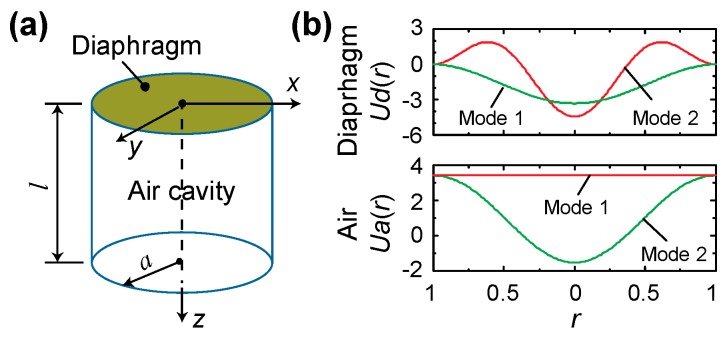
Introduction of the studied problem. (**a**) Schematic of a circular clamped diaphragm backed by a cylindrical air cavity, which has a rigid sidewall and bottom. (**b**) The mode shapes of the first two axisymmetric modes of the diaphragm (top) and air cavity (bottom).

To fully capture the acoustic–structural interaction, various analytical mechanics models have been developed, which are categorized in terms of how the cavity is modeled: i) using the Reynolds equation for thin viscous fluid films (also referred as squeeze film damping) [12,13,14,15,16], or ii) using the sound wave equation where the viscous term is usually neglected [17,18,19,20,21,22,23,24,25,26]. Following the second approach, but in the context of dynamic pressure sensors, we have previously developed an analytical model to investigate how the static mechanical sensitivity $SS_{c}$ and fundamental natural frequency $f_{c1}$ change when the cavity length *l* continuously decreases [27]. Note subscripts *d*, *a*, and *c* are used throughout this paper to denote the diaphragm, the air, and the coupled system of the air-backed diaphragm, respectively. Using a circular clamped diaphragm backed by a cylindrical air cavity as a representative configuration, as shown in Figure 1a, the diaphragm was modeled as a thin-plate with in-plane tension and the air cavity was described using a wave equation in terms of the velocity potential. A geometric compatibility condition is assumed at the diaphragm–air interface, i.e., continuous displacement/velocity in the normal direction.

A key finding from the analytical model was that the air cavity changes the effective stiffness and mass of the diaphragm. A summary is provided here. Assuming that for small sensors, the diaphragm’s radius *a* is much smaller than the wavelength *λ* (i.e., a≪λ), the transverse (z-direction) displacement excitation at the top of the air cavity can be expressed as $w_{a}\left(r,\theta ,t\right)=\sum_{n=0}^{\infty }W_{a,n}U_{a,n}\left(r\right)\Theta_{0}\left(\theta \right)e^{j\omega t}$, where the displacement is assumed to be axisymmetric (hence $\Theta_{0}$ is a constant $\Theta_{0}\left(\theta \right)=1/\sqrt{2\pi }$), $U_{a,n}\left(r\right)$ are the mode shape functions in the radial direction (shown in the bottom of Figure 1b for the first two modes along with the first two axisymmetric diaphragm modes at the top), and $W_{a,n}$ are the modal coefficients to be determined. The corresponding reaction pressure from the air to the backside of the diaphragm can be expressed as $p_{R}\left(r,\theta ,t\right)=\sum_{n=0}^{\infty }P_{Ra,n}U_{a,n}\left(r\right)\Theta_{0}\left(\theta \right)e^{j\omega t}$, where $P_{Ra,n}$ are the coefficients to be determined. When the cavity length is much smaller than the wavelength (*l* ≪λ), it can be found that $H_{a,n}=\left(P_{Ra,n}/p_{0}\right)/\left(W_{a,n}/a\right)$ is equal to γa/l for the first mode (*n* = 0), where *γ* is the ratio of specific heat (1.4 for air), and $p_{0}$ is the ambient pressure. The effect of this mode is to increase the effective stiffness of the diaphragm, with the air cavity serving as an equivalent spring. For the second- or higher-order modes (n≥1), $H_{a,n}\approx -M_{a,n}\Omega_{a}^{2}$, where $M_{a,n}=-\left(\gamma /\beta_{n}^{2}\right)\left(a/l\right)$, $\Omega_{a}=\omega a/c_{0}$ is the normalized frequency, $c_{0}$ is the speed of sound in air, and $\beta_{n}$ are the roots of the characteristics equation ($\beta_{1}$ = 3.832, $\beta_{2}$ = 7.016). The effects of these modes are to increase the effective mass of the diaphragm. As *l* decreases, both the effective stiffness from the first mode and the effective mass from the second- and higher-order modes increase, as they scale with 1/l. Note that if the diaphragm moves like a piston, only the first mode will be excited, and thus, the closed air cavity can be modeled simply as a lumped spring element. However, when the diaphragm’s circumference is clamped, the higher-order modes will be excited. As such, there is an interplay between the stiffness and mass effects as *l* decreases. The following conclusions were reached for the air-backed diaphragm from the analytical model, which was validated using finite element method (FEM) simulations in COMSOL: (i) As *l* decreases, $SS_{c}$ decreases monotonically. (ii) As *l* decreases, $f_{c1}$ shows a three-stage trend: it increases in the long-cavity range, reaches a plateau value in the medium-cavity range, and decreases in the short-cavity range. It is important to note that the SDOF lumped model cannot explain these findings.

A related work to experimentally study the effects of an air cavity was carried out by Gorman et al. [18]. A test rig was developed consisting of an aluminum plate (380 μm in thickness and 76 mm in diameter) backed by a cylindrical cavity with a length of 81 mm or 255 mm. The diaphragm was excited by a shaker, its mode shape was observed by Chladni sand method, and two microphones were inserted into the sidewall and base to measure the acoustic pressure. Consistent with the analytical and numerical results, a key conclusion from the study was that strong acoustics–structure interaction exists when the fundamental natural frequencies of the two sub-systems (the diaphragm in vacuo and the closed cavity) are close. However, a comprehensive experimental study is still lacking to systemically investigate the effects of changing air cavity length, particularly in the context of dynamic pressure sensor development.

In this study, as a follow-up study to the analytical model [27], the goal was to conduct the first experimental measurements to systemically validate the effects of the air cavity on $SS_{c}$ and $f_{c1}$ when *l* continuously decreased, serving as a fundamental guideline for the design and development of dynamic pressure sensors. A parametric study was conducted by varying the in-plane tension, which is a common issue to deal with in sensor fabrication. The experiment results were also validated by comparing them with analytical and numerical simulations.

## 2. Methods

**Figure 2:**
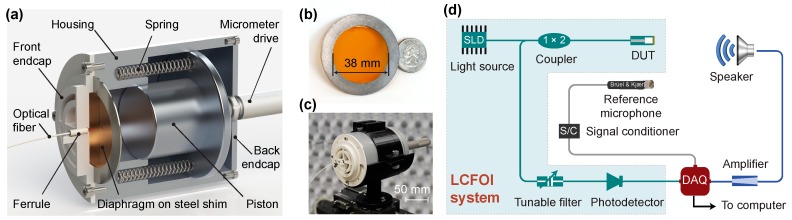
Experimental design to investigate the acoustics–structure interaction between a diaphragm and its backing cavity. (**a**) Schematic of the setup to vary the backing air cavity length. (**b**) Photo of the polyimide diaphragm with its edge glued to a circular steel shim (next to a dime). (**c**) Photo of the integrated device under test (DUT) mounted on a stage. (**d**) Schematic of the measurement system including a low-coherence fiber-optic interferometer (LCFOI) system in the shaded area. DAQ: Data acquisition card, SLD: superluminescent diode.

The system used to experimentally investigate the effects of air cavity on sensor performance is illustrated in Figure 2. As shown in the cross-section view (Figure 2a), the main components included a machined aluminum housing (outside diameter (OD): 76 mm, length: 86 mm), a machined aluminum sliding piston, a circular polyimide diaphragm clamped to a steel shim (inside diameter (ID): 38 mm, OD: 54 mm, thickness: 0.82 mm), and two endcaps (front and back). The housing had an internal cavity with a diameter of 38 mm and a length of 30 mm. The piston had a clearance fit with the housing and was pushed against two springs using a micrometer drive (Newport SM-50 Vernier micrometer, 10-μm increment) attached to the back endcap, providing a turning mechanism to vary the length of the backing air cavity. The front endcap was fabricated using a 3D printer (Ultimaker 3 Extended) using a polylactic acid (PLA) filament. Sound could reach the diaphragm through the large openings on the front endcap. A ceramic ferrule (Thorlabs CFLC128, ID: 128 μm) was press-inserted into a center hole on the front endcap, which was used to guide an optical fiber for detecting the center displacement of the diaphragm.

Thermal stress was generated to adjust the in-plane tension in the polyimide diaphragm (thickness: 50.8 μm). The diaphragm and the steel shim were preheated in an oven (Thermo Scientific, Model 6050) to a temperature *T*. Then, the shim was smeared with 2-h curing epoxy (FIS T120023C250) and seated on top of the diaphragm in the oven. After being heated for 2 h, the diaphragm was taken out and cooled down to room temperature *T*_0_ (~22 °C), which generated in-plane tension in the diaphragm that was proportional to the temperature difference $\Delta T=T-T_{0}$ (referred to as the shrinkage temperature hereafter); see Figure 2b for the photo of the diaphragm.

Next, with the diaphragm attached, the shim was placed in the groove on the front endcap (see Figure 2a) and pressed against the housing with four screws, aligning the diaphragm with the internal cavity. A square-shaped aluminum plate (2.3 mm × 2.3 mm × 18 μm) was glued to the diaphragm center as a reflection mirror. A cleaved optical fiber (Corning SMF-28e) was inserted into the ferrule. The distance between the fiber tip and the aluminum plate was monitored using a fiber optic interrogator (Micron Optics Si155-04-ST). When it reached ≈60 μm, the fiber was fixed to the ferrule using a UV-curable epoxy (Dymax OP-4-20632); see Figure 2c for a photo of the whole integrated device.

The center displacement of the diaphragm subject to acoustic pressure stimuli was measured using a low-coherence fiber-optic interferometer (LCFOI) system [5], illustrated in the shaded area of Figure 2d. Compared with the conventional laser-based interferometer, LCFOI has one particular advantage, namely less susceptibility to wavelength and power fluctuations, which makes it ideal for measuring the displacement down to the nanometer scale or smaller. The light from a broadband superluminescent diode (SLD) (Thorlabs S5FC1018S, center wavelength 1310 nm, 40 BW, 30 mW) was delivered via a 1 × 2 fiber-optic coupler (Gould Fiber Optics, 50:50) to the Fabry–Pérot (FP) interferometer of the sensor head (i.e., the sensing interferometer). The reflected light was then sent to an FP-tunable filter (Micron Optics FFP-TF2, as the reference interferometer) via the same 1 × 2 coupler. The output light from the tunable filter was coupled into the photodetector (Newport, model 2011) and converted into an electric signal. For the whole measurement system shown in Figure 2d, a half-inch condenser microphone (Brüel & Kjær, model 4191) was used as a reference sensor, from which the input pressure could be obtained. A data acquisition card (DAQ, National Instruments, model USB-6366) was connected to a computer to output the sound stimulus through a speaker (Adam A8x) and to acquire the signals from the reference microphone and the fiber-optic acoustic sensor. The sampling frequency was 200 kHz, compared with the test frequency of 500 Hz–2 kHz.

To obtain the mechanical sensitivity in terms of the center displacement per unit pressure, we followed our previous method detailed in Dong et al. [28] based on the interchangeability of the sensing interferometer and the reference interferometer in an LCFOI system. When the electric output of the LFFOI system was converted to the diaphragm displacement, it could be combined with the measured pressure stimuli from the reference microphone to obtain the frequency response function, i.e., the mechanical sensitivity as a function of frequency.

## 3. Results

### 3.1. Finite Element Simulation

**Figure 3:**
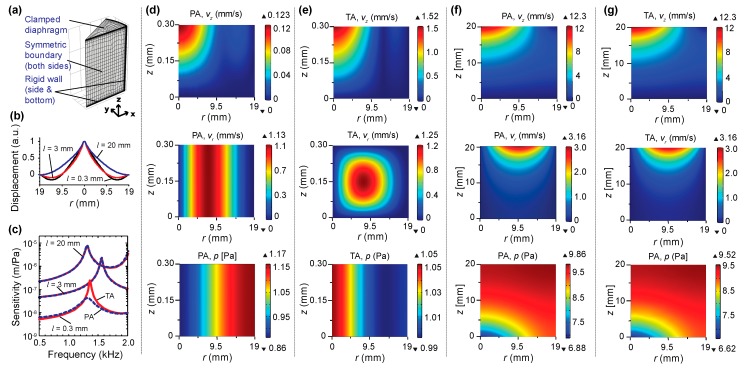
Finite element model (FEM) model and simulation results. (**a**) FEM model in COMSOL. (**b**) Mode shape (fundamental) for the three cavity lengths (*l* = 0.3 mm, 3 mm, and 20 mm) obtained using an eigenfrequency study in the COMSOL Pressure Acoustics (PA) module. (**c**) Mechanical sensitivity as a function of frequency for the three cavity lengths obtained using (PA, solid line) and the Thermoviscous Acoustics (TA, dashed line) module. (**d**–**g**) Field distribution of the magnitudes of the velocity in the normal/z-direction (top), the velocity in the tangential/radial direction (middle), and the acoustic pressure (bottom) for four cases: (**d**): PA, *l* = 0.3 mm; (**e**) TA, *l* = 0.3 mm; (**f**) PA, *l* = 20 mm; and (**g**) TA, *l* = 20 mm. The frequency for all cases in (**d**–**g**) was 1.3 kHz.

In the analytical model [27], the thermoviscous effect of the backing air cavity was not considered. In this follow-up study, the cavity length was varied from 0.2 mm to 30 mm. For comparison, the viscous boundary layer thickness was 69.3 μm at 1 kHz, room temperature, and standard pressure [29]. To illustrate the size effect and provide a validation for the experimental study, an FEM model was developed using the Pressure Acoustics (PA) module or the Thermoviscous Acoustics (TA) module, in combination with the acoustics–structure interaction module in COMSOL 5.3a. Note that PA is based on the Helmholtz equation where it is assumed to be lossless, while TA is based on the linearized Navier–Stokes equations where the thermoviscous effects are considered. Taking advantage of the symmetry, as shown in the FEM model (Figure 3a), only a 30° segment was modeled: the air cavity was meshed using second-order brick elements, the shell was meshed using quadrilateral shell elements, and the boundary layer meshes were added near the walls. The parameters for the diaphragm are listed in Table 1, which were used in the simulation and served as the nominal values for the experiments detailed in Section 3.2. Note that the effective coefficient of thermal expansion (CTE) *μ* is the difference between the CTE values of the stainless shim and polyimide diaphragm (*μ* = 12.8 × 10^−6^ K^−1^). For a shrinkage temperature Δ*T*, the in-plane tension introduced in the circular clamped diaphragm with thickness *h* and Young’s modulus *E* is EμΔTh, which is equal to 114 N/m for Δ*T* = 80 °C.

As shown in Figure 3b, the mode shape of the fundamental mode changed with the cavity length due to the acoustics–structure interaction between the diaphragm and the backing air cavity, which affected its response to external pressure stimuli. The mechanical sensitivity was obtained by carrying out a frequency-domain study in COMSOL, where a uniform pressure ($p=p_{0}e^{j\omega t}$, $p_{0}$ = 1 Pa) was applied to the external surface of the diaphragm. The displacement of the diaphragm center was extracted to obtain the mechanical sensitivity as the frequency was swept from 500 Hz to 2 kHz for three different cavity lengths (*l* = 0.3 mm, 3 mm, and 20 mm), as shown in Figure 3c. As the cavity length decreased, $SS_{c}$ decreased; however, $f_{c1}$ increased first but then decreased in the short-cavity-length range. These results are consistent with the results from the analytical model [27]. In terms of the difference between the simulation results obtained using TA and PA, when the cavity length was long (*l* = 3 mm or 20 mm), the difference was negligible. Only when the cavity length was closer to the boundary layer thickness (e.g., *l* = 0.3 mm), there was noticeable discrepancy, particularly near the resonance where the damping effect dominated. This discrepancy was due to the nature of the underlying governing equations: only the normal velocity was compatible at the acoustics–structure interface in the PA case, whereas both normal and tangential velocities were compatible in the TA case. Figure 3d–g shows the velocity (normal and tangential) and pressure fields for the excitation frequency of 1.3 kHz. When the cavity length was small (*l* = 0.3 mm), the normal velocity (z-direction) had similar profiles (Figure 3d,e top), but the magnitude was smaller in the PA case (Figure 3d top) than in the TA case (Figure 3e top). As for the tangential velocity, it was uniform in the depth direction in the PA case (Figure 3d middle), whereas there were clearly boundary layers adjacent to the solid walls in the TA case (Figure 3e middle). As a result, there were significant differences in their pressure fields (Figure 3d,e bottom). When the cavity length was long (*l* = 20 mm) and much larger than the boundary layer thickness, the difference was negligible, as evidenced by the similarity of the velocity and pressure fields shown in Figure 3f,g. To ensure consistency, the COMSOL TA simulation was used for comparison with the experimental results in the next section.

### 3.2. Experimental Results

**Figure 4:**
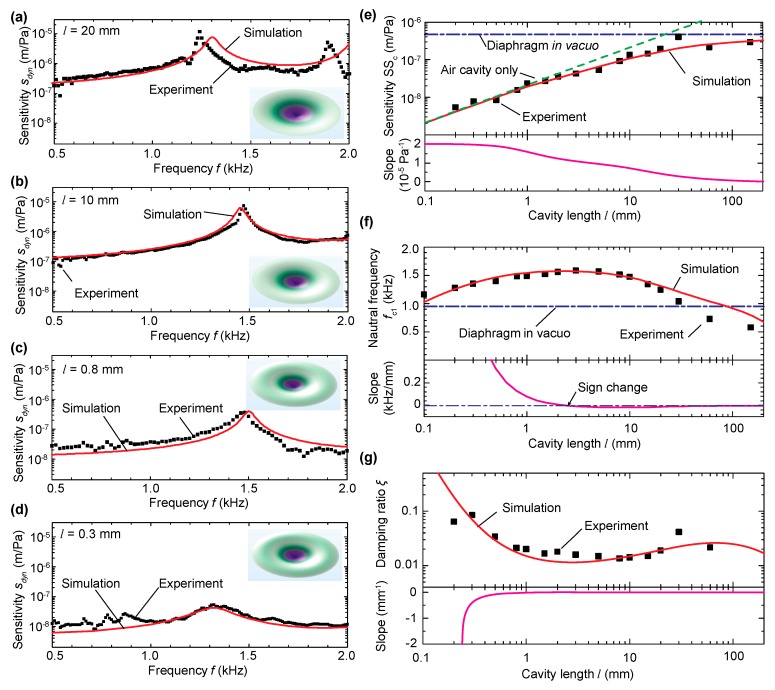
Experimental results in comparison with the simulations. (**a**–**d**): Frequency response function (i.e., mechanical sensitivity as a function of frequency) for different cavity lengths (*l* = 20 mm, 10 mm, 0.8 mm, and 0.3 mm), where the simulation results were obtained using the COMSOL Thermoviscous Acoustics module. The insets show the mode shapes for the fundamental mode. (**e**–**g**): mechanical sensitivity (**e**), natural frequency (**f**), and damping ratio (**g**) as a function of cavity length. The curves in the bottom of (**e**–**g**) show the slopes of the simulated data.

The device fabricated in Section 2 and the FEM model in Section 3.1 were used to parametrically study the effects of the air cavity on the performances (sensitivity and natural frequency) of acoustic pressure sensors. Figure 4a–d shows the measured dynamic mechanical sensitivity $s_{dyn}$ of the center diaphragm displacement in response to acoustic pressure stimuli as a function of frequency *f* when *l* decreased from 20 mm to 10 mm, 0.8 mm, and 0.3 mm, along with the FEM simulation results. For simplicity, the mechanical sensitivity can be approximated as a function of frequency *f* via an equivalent single-degree-of-freedom system as:

(1) $$s_{dyn}\left(f\right)=\frac{SS_{c}}{\sqrt{{\left[1-{\left(f/f_{c1}\right)}^{2}\right]}^{2}+{\left[2\xi \left(f/f_{c1}\right)\right]}^{2}}},$$

where ξ is the damping ratio. The measured and the simulated sensitivity data can be curve-fitted to extract $SS_{c}$, $f_{c1}$, and ξ, which are shown in Figure 4e–g as a function of *l*.

As shown in Figure 4e, the mechanical sensitivity $SS_{c}$ increased with increasing cavity length due to the stiffness effect (i.e., the longer the length, the softer the cavity), and asymptotically approached the value $1/k_{d}$ in vacuo (i.e., when the effects of the backing air cavity were neglected), where $k_{d}$ is the effective stiffness of the diaphragm. The stiffness due to the air cavity alone $k_{a}$ can be calculated from the adiabatic process as $k_{a}=l/\left(\eta \gamma p_{0}\right)$, where η is a factor to account for the non-uniform deflection of the diaphragm contributing to the change of air volume. η can be calculated using $\eta =\int_{r=0}^{a}u_{s}\left(r\right)2\pi rdr/\left[u_{s}\left(r=0\right)\pi a^{2}\right]$, where $u_{s}\left(r\right)$ is the static deflection of the diaphragm under uniform pressure (η = 0.33 for the circular clamped diaphragm). Combining the above two yields the sensitivity of the air-backed diaphragm $SS_{c}=1/\left(k_{d}+k_{a}\right)$, as indicated by the three curves in Figure 4e top. In other words, the measured static sensitivity was consistent with the theory that the stiffness of the air-backed diaphragm is the sum of those of the diaphragm in vacuo and the air cavity. The bottom of Figure 4e shows the slope of $SS_{c}$ with respect to *l*, which starts from an almost constant value and then asymptotically decreased to zero.

Different from the monotonic relationship between $SS_{c}$ and *l*, the fundamental natural frequency $f_{c1}$ showed a three-stage trend as the cavity length decreased (as shown in Figure 4f top): it increased in the long-cavity-length range, reached a plateau value in the medium-cavity-length range, and decreased in the short-cavity-length range. The slope shown in the bottom of Figure 4f shows a sign change at the cavity length indicated by the arrow, corresponding to the maximum $f_{c1}$. As derived in Liu et al. [27], the behavior of an air-backed diaphragm in the short-cavity-length range can be modeled using two air modes and two diaphragm modes. The effects of the first and second air modes are to increase the stiffness and mass, respectively, both of which scale with 1/l. The fundamental mode of the air-backed diaphragm can be considered a hybridization of the two modes. As *l* decreases, the air cavity becomes stiffer, and the contribution from the second mode increases. The analytical derivation further shows that $f_{c1}$ scales with $\sqrt{l}$ in the short cavity length range, which explains the trend in Figure 4f for *l* < 1 mm.

The overall trend regarding how the damping ratio changes with cavity length is opposite to that of the natural frequency with some exceptions. In the very short cavity length range (*l* < 0.2 mm), as indicated by the simulation data in Figure 3g, the damping ratio increased sharply due to the enhanced viscous damping as the cavity length decreased. In the long-cavity-length range (*l* > ≈80 mm), the damping ratio started to drop as the cavity length increased. This was due to the fact that the fundamental mode in this range was an acoustic mode. Note that for dynamic pressure sensors, damping can be tuned post-fabrication by changing the design of the holy plate underneath the diaphragm [30,31]. As such, the focus of this study was on the sensor sensitivity and the fundamental natural frequency, which are the two important metrics in sensor design.

**Figure 5:**
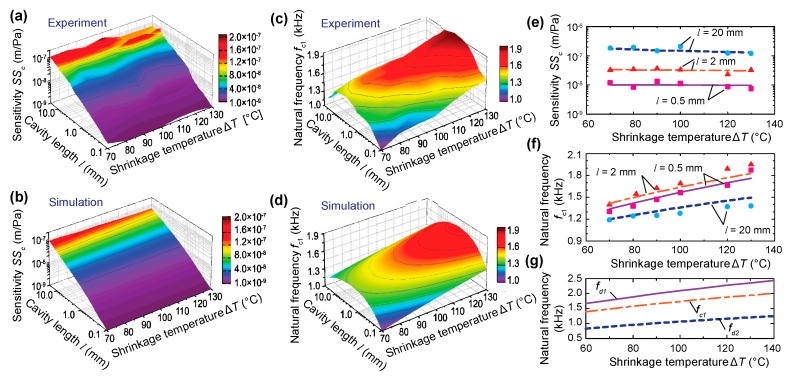
Parametric study by changing the in-plane tension in the diaphragm via thermal expansion and cooling. (**a**,**b**) Contour plots of sensitivity as a function of cavity length *l* and shrinkage temperature Δ*T* ((**a**) experiment, (**b**) simulation). (**c**,**d**): same as (**a**,**b**) but for the natural frequency. (**e**,**f**): Mechanical sensitivity (**e**) and natural frequency (**f**) as a function of Δ*T* for three different cavity lengths: 0.5 mm, 2 mm, and 20 mm (scatter points: experiment, lines: simulation). (**g**) Simulated natural frequencies of the first two modes of the diaphragm in vacuo (denoted as $f_{d1}$ and $f_{d2}$) and the first fundamental frequency of the air-backed diaphragm with *l* = 2 mm, denoted as $f_{c1}$.

A parametric study was further carried out to validate the above finding when the in-plane tension was varied by changing the shrinkage temperature Δ*T* from 70 °C to 130 °C (the corresponding in-plane tension varied from 100 N/m to 186 N/m). As shown in Figure 4a–d, both the sensitivity $SS_{c}$ and the fundamental natural frequency $f_{c1}$ are plotted as a function of the air cavity length *l* and shrinkage temperature Δ*T*. Overall, there was a good agreement between the experimental and simulation results, and similar three-stage trends in terms of the effect of cavity length were observed for all tested values of Δ*T*, validating the results in the analytical model [27]. In the studied cavity length range, the air cavity was much stiffer than the diaphragm. As such, for a fixed cavity length, increasing Δ*T* increased the stiffness of diaphragm in vacuo but the stiffness of the air-backed diaphragm was largely unchanged, as shown in Figure 5c for three representative values of *l* (0.5 mm, 2 mm, and 20 mm). However, increasing Δ*T* increased the fundamental frequency $f_{c1}$, as shown in Figure 5d for the same values of *l*. As discussed in Liu et al. [27], the plateau frequency in the medium cavity length range was between the first and second natural frequencies $f_{d1}$ and $f_{d2}$ of the diaphragm in vacuo. As shown in Figure 5g, when the in-plane tension increased, both $f_{d1}$ and $f_{d2}$ increased. As a result, $f_{c1}$ of the air-backed diaphragm increased accordingly.

The discrepancy between the experimental data and simulation results could be attributed to several factors. The first was the nonlinearity and hysteresis of the tunable filter of the optical detection system when a bias voltage was applied to the reference filter. Second, although the testing was conducted in a room with absorbing foams on the sidewalls and ceiling, it was not an anechoic chamber per se; there were some unwanted echoes during the testing. Third, the front cap with the fiber optic probe may have disturbed the incident sound wave. The fourth was the variation of the point mass added to the diaphragm center. Lastly, the thermal stress in the diaphragm may not have been consistently controlled due to the fluctuation of room temperature and relaxation of the diaphragm.

## 4. Conclusions

A comprehensive experimental study was carried out to systemically investigate how the diaphragm in a dynamic pressure sensor interacts with the backing air cavity and affects the sensor performance. Large-scale dynamic pressure sensors using circular clamped polyimide diaphragms were fabricated and characterized when the length of the backing cavity and the in-plane tension in the diaphragm were varied. The experimental results compared well with the FEM simulations and validated the previous findings from the analytical model [27] on the static mechanical sensitivity and the fundamental natural frequency $f_{c1}$. As the cavity length *l* decreased, $SS_{c}$ decreased monotonically. When *l* was below a critical value, the stiffness of the air cavity dominated that of the diaphragm. For $f_{c1}$, it showed a three-stage trend as *l* decreased: increasing in the long-cavity-length range, reaching a plateau value in the medium-cavity-length range, and decreasing in the short-cavity-length range. 

These experimentally validated findings have a profound implication on how to model a simple yet common structure of an air-backed diaphragm. Traditionally, the backing air cavity has been modeled as an effective spring element *k_a_*, as taught in acoustics [11] or sensor design [32] textbooks. However, this approach of modeling cannot capture the full acoustics–structure interaction between the diaphragm and the backing air cavity correctly, particularly when the cavity length is very short. The traditional lumped model will always predict an increased fundamental natural frequency as the cavity length becomes shorter. However, as demonstrated in this paper, the interaction is more complex. The backing air cavity affected both the effective stiffness and mass of the diaphragm. Shorter air cavity resulted in larger *k_a_*, and thus increased the diaphragm’s effective stiffness. A shorter air cavity also increased the effective mass, although the total mass of the air enclosed in the cavity was smaller. The combination of these two effects led to a counterintuitive phenomenon in the short-cavity-length range where a shorter cavity length caused the fundamental natural frequency to decrease. 

These findings also serve as important guidelines for practically designing dynamic pressure sensors, particularly for miniaturized sensors where the cavity must be made short to reduce the overall sensor size. If the configuration is a uniform circular clamped diaphragm backed by a cylindrical air cavity, the formula provided in Liu et al. [27] would be useful to determine whether the cavity length falls within the long-, medium-, or short-cavity-length regimes, and estimate the sensitivity and fundamental natural frequency. For more complex configurations, FEM simulations can be used to guide the sensor design.

## Figures and Tables

**Table 1:** Properties of the polyimide diaphragm.

Young’s modulus *E_d_*	2.2 GPa
Poisson’s ratio *v*	0.3
Density *ρ*_d_	1290 kg/m^3^
Radius *a*	19 mm
Thickness *h_d_*	50.8 μm
Expansion temperature Δ*T*	80 °C
Coefficient of thermal expansion *μ*	12.8 × 10^−6^ K^−1^
Rayleigh damping *α* (*β* = 0)	500 s^−1^
Point mass at the center $m_{p}$	3 mg
